# Visualizing the Growth and Division of Rat Gut Bacteria by D-Amino Acid-Based *in vivo* Labeling and FISH Staining

**DOI:** 10.3389/fmolb.2021.681938

**Published:** 2021-05-28

**Authors:** Ru Chen, Jia Song, Liyuan Lin, Jie Liu, Chaoyong Yang, Wei Wang

**Affiliations:** ^1^ Department of Digestive Diseases of Huashan Hospital and Institutes of Biomedical Sciences, Fudan University, Shanghai, China; ^2^ Institute of Molecular Medicine, Renji Hospital, Shanghai Jiao Tong University School of Medicine, Shanghai, China; ^3^ The MOE Key Laboratory of Spectrochemical Analysis and Instrumentation, Key Laboratory for Chemical Biology of Fujian Province State Key Laboratory of Physical Chemistry of Solid Surfaces, Department of Chemical Biology, College of Chemistry and Chemical Engineering, Xiamen University, Xiamen, China

**Keywords:** metabolic labeling, gut microbiota, rat, D-amino acid-based probes, FISH, bacterial division

## Abstract

Rat is a widely used mammalian model for gut microbiota research. However, due to the difficulties of individual *in vitro* culture of many of the gut bacteria, much information about the microbial behaviors in the rat gut remains largely unknown. Here, to characterize the *in situ* growth and division of rat gut bacteria, we apply a chemical strategy that integrates the use of sequential tagging with D-amino acid-based metabolic probes (STAMP) with fluorescence *in situ* hybridization (FISH) to rat gut microbiota. Following sequential gavages of two different fluorescent D-amino acid probes to rats, the resulting dually labeled gut bacteria provides chronological information of their *in situ* cell wall synthesis. After taxonomical labeling with FISH probes, most of which are newly designed in this study, we successfully identify the growth patterns of 15 bacterial species, including two that have not been cultured separately in the laboratory. Furthermore, using our labeling protocol, we record *Butyrivibrio fibrisolvens* cells growing at different growth stages of a complete cell division cycle, which offers a new scope for understanding basic microbial activities in the gut of mammalian hosts.

## Introduction

The compositions and activities of the microbial community in the gut reflect the co-evolution between the host and gut microbes (Marchesi et al., 2016). The intestines represent an attractive niche with stringent conditions, rich in nutrients and microbes. Meanwhile, the microbial community in the gut conveys significant benefits to the host, such as the enzymatic capacities to break down dietary fibers (Gill et al., 2006), production of a great variety of metabolites (Nicholson et al., 2012), and the establishment of a barrier against invading pathogens (Boulangé et al., 2016). Gut microbiotas differ greatly among mammalian hosts (Ley et al., 2008). The conventional approach of *in vitro* culture limits our comprehension of the gut microbiota to the characterization of cultivable microbes (Browne et al., 2016). Increasingly updated high-throughput sequencing technologies have significantly promoted our understanding of the composition and diversity of host-associated microbial populations that are not investigable by culture-dependent methods. Nonetheless, their growth status and indigenous activities have been highly challenging to study.

Recently, we developed an integrative strategy which used sequential tagging with D-amino acid-based metabolic probes (STAMP) together with fluorescence *in situ* hybridization (FISH) for directly probing and visualizing the *in vivo* microbial growth in the mouse gut (Lin L. et al., 2020). As the first mammalian species domesticated for scientific research, rats are commonly used in the research of cardiovascular diseases (Kräker et al., 2020), behavioral and neurological disorders (Zhang et al., 2017; Vuralli et al., 2019), metabolic diseases (Kowluru, 2020), cancer (Seluanov et al., 2018) and autoimmune diseases (Zhong et al., 2018), as well as gut microbiotas (Tomas et al., 2012). Using techniques such as 16S rDNA sequencing, quantitative polymerase chain reaction (qPCR) and FISH (Qin et al., 2010), researchers have found that rats have gut microbiotas that are compositionally more similar with human’s than mice have (Manichanh et al., 2010). Indeed, another study suggested that humanized rat models had a more similar *Firmicutes* to *Bacteroidetes* ratio with human donors, and thus could represent the human donor better than the mouse models could (Wos-Oxley et al., 2012). Moreover, sequencing results of mouse and rat gut microbiotas also showed the compositional differences between the two systems (Manichanh et al., 2010). Therefore, it is still of great value to explore cellular microbiology, such as bacterial growth and division patterns, of the rat gut microbiota for further understanding the physiological and pathological functions performed by these gut microbes.

Here, we propose the integrative use of STAMP + FISH labeling to investigate rat’s gut microbiota. After sequentially administered two fluorescent D-amino acid-based probes (FDAA) containing different fluorophores to SD rats by gavages, we identified a panel of bacterial species by FISH staining, and imaged their *in situ* growth and multiplication processes using their FDAA labeling signals. It presents an easy-to-operate and efficient approach to address basic microbiology questions of gut “dark matter” in rat, showcasing the applicability of this integrative protocol in studying the gut microbiotas in different mammalian hosts.

## Results

### *In Vivo* Sequential Fluorescent D-amino Acid Labeling of Rat Gut Microbiota

**FIGURE 1:**
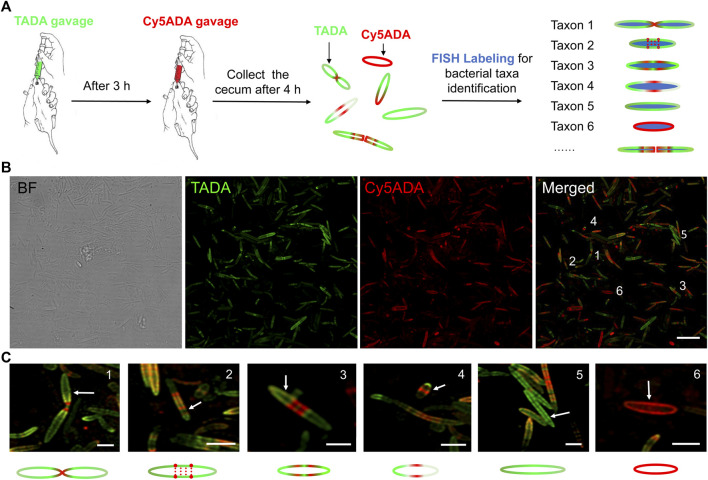
Schematic illustration of STAMP and FISH labeling strategy, and the two-color fluorescence imaging of the labeled rat gut microbiota. **(A)** TADA and Cy5ADA were given to SD rats by gavage at an interval of 3 h. Four hours after the second gavage, their cecal microbiotas were collected and imaged. Bacterial species of interest were then separately stained and visually identified using corresponding FISH probes. **(B)** Two-color fluorescence imaging of the gut bacteria sequentially labeled by TADA (green) and Cy5ADA (red). Representative images from at least three independent experiments are shown. BF, bright field. Scale bar, 10 μm. **(C)** Zoomed in views of the bacteria from the merged image above. The green and red colors indicated the distinct growth patterns of different bacteria. Scale bars, 2 μm.

FDAAs can be covalently incorporated into bacterial peptidoglycan (PGN) by the catalysis of endogenous D,D or L,D-transpeptidases of the bacteria, which provides a versatile strategy for examining PGN synthesis during the bacterial growth and division (Kuru et al., 2019). Recently, it has been demonstrated that multiple FDAAs could be chronologically incorporated into bacterial PGN *in vivo* (Hudak et al., 2017; Lin L. et al., 2020). Here, to reveal the growth and division patterns of gut bacteria in rats, we used the STAMP protocol to label their gut microbiotas with two different FDAAs: TAMRA-amino-D-alanine (TADA) and Cy5-amino-D-alanine (Cy5ADA), containing TAMRA (tetramethylrhodamine) or Cy5 (Cyanine 5) on their side chains, respectively (Figure 1A).

In previously reported mouse microbiota labeling, the FDAAs were given at an interval of 3 h (Lin L. et al., 2020). Considering that the gastric emptying and intestinal motility time of rats are comparable to mice (Lu et al., 2017), here we adopted a similar *in vivo* labeling protocol. The collected cecal microbes after STAMP showed strong dual-color labeling, and the green and red signals observed by confocal microscopy revealed the PGN synthesis processes of the labeled gut bacteria (Figure 1B). Flow cytometry also showed high labeling coverages of the two FDAAs (Supplementary Figure S1), indicative of an efficient *in vivo* labeling of the rat microbiotas.

The two-color fluorescence imaging presented a high morphological diversity of rat gut microbes with strong FDAA labeling throughout the cells (Figure 1B). The different distributions of labeling signals indicated distinct growth and division patterns: septum synthesis division (Figure 1C, No.1), segmented/continuous diffuse synthesis (No. 2 and 3), and asymmetric/polar synthesis (No. 4), to name a few. We also observed bacteria with only TAMRA or Cy5 signals (No. 5 and 6), which suggested that these cells might have different growth rates during the two labeling stages.

### Identification of Growth Patterns of Individual Species

After obtained the resourceful imaging of the FDAA-labeled rat gut microbiota, we set out to identify the growth patterns of bacteria on the species level. FISH has proved to be a powerful method for visualization and identification of microbes in complex environments (Sunde et al., 2003). To determine the taxonomic composition of the microbiota, we performed both 16S rDNA and metagenomic sequencing (Supplementary Figure S2 and Supplementary Table S1). Based on the results of metagenomic sequencing, 25 species with relatively high abundances (>0.12%, covering ∼35% of the total population, Supplementary Table S1) were selected and taxonomically labeled with corresponding FISH probes (Supplementary Tables S2,S3). The FISH sequences were either based on previous reports or designed in this study using an algorithm that was previously developed and recently optimized (Kong et al., 2010; Lin L. et al., 2020). To verify the specificities of the newly designed FISH probes, the labeling patterns and cell morphologies of each stained species were carefully examined. Only the species with highly consistent patterns and morphologies (Supplementary Figure S3) were reported herein. Among the 25 FISH probes, we were able to confirm the specificities of 15 sequences based on the criteria (listed in Supplementary Table S2).

**FIGURE 2:**
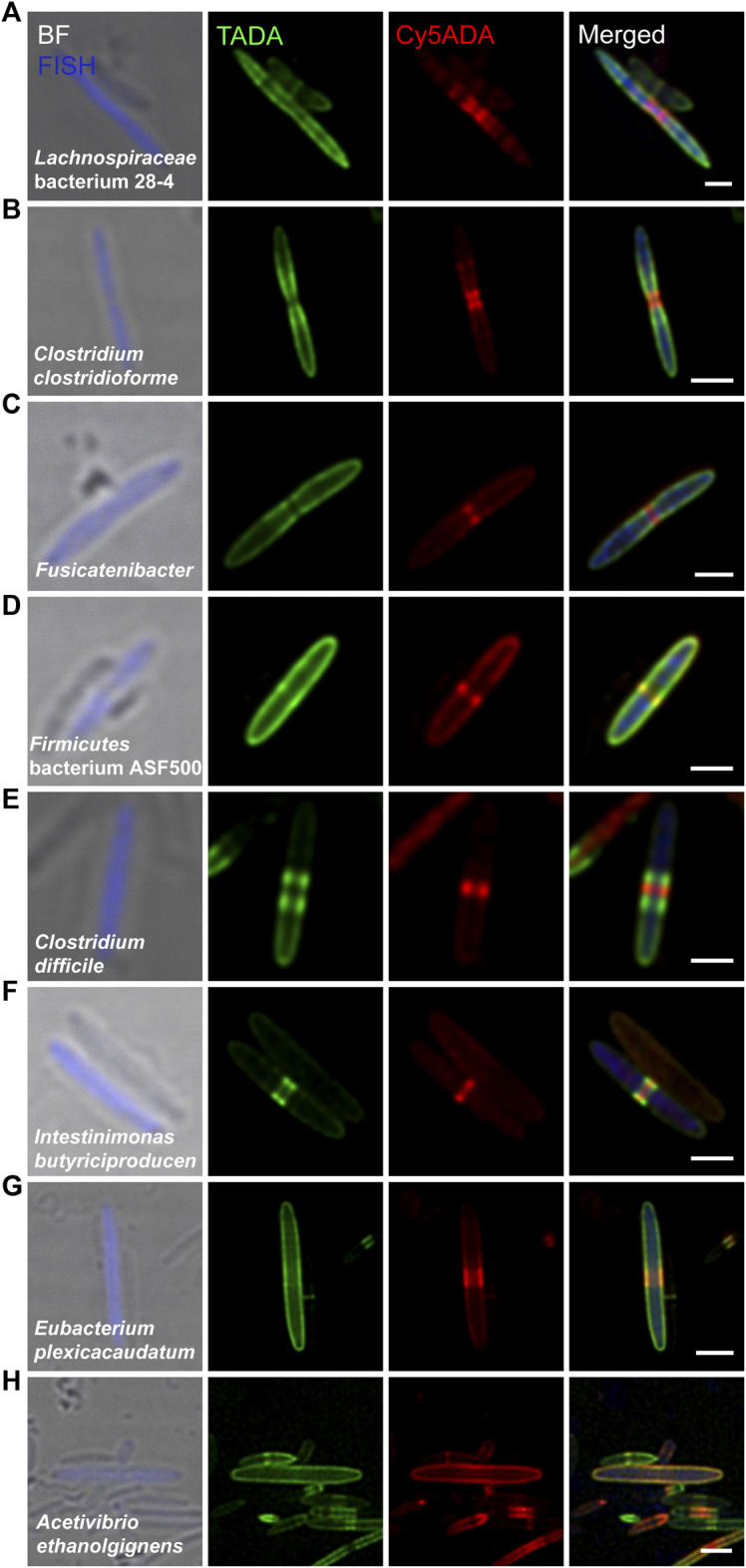
Confocal fluorescence imaging of eight FDAA-labeled and FISH-stained Gram-positive species in rat cecal microbiota. The cecal microbiotas of rats that received sequential labeling of TADA (green) and Cy5ADA (red) were stained by different FISH probes (blue) targeting corresponding species. **(A–H)** Representative images of FDAA-labeled bacteria in eight Gram-positive species. For each species, bacterial image representative of consistent FDAA-labeling patterns from at least three independent FISH experiments is shown. Scale bars, 2 μm.

**FIGURE 3:**
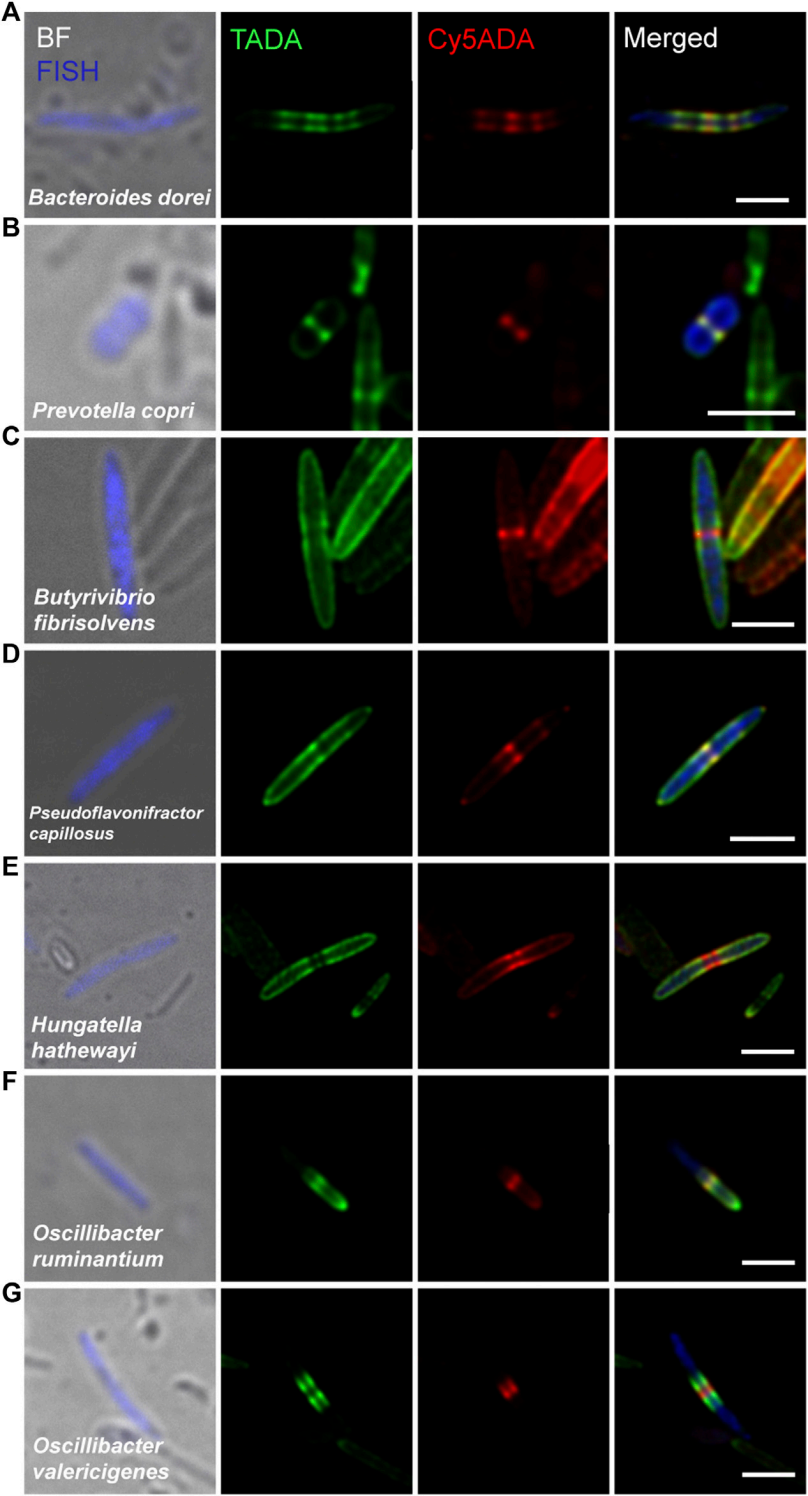
Confocal fluorescence imaging of seven FDAA-labeled and FISH-stained Gram-negative species in rat cecal microbiota. The cecal microbiotas of rat received sequential labeling of TADA (green) and Cy5ADA (red) was stained by different FISH probes (blue) targeting corresponding species. **(A–G)** Representative images of FDAA-labeled bacteria in seven Gram-negative species. Scale bars, 2 μm. Photographs of bacteria, which showed consistent labeling pattern in each species from at least three independent FISH experiments, are shown.

These bacteria were from 14 genera of ten families, containing eight Gram-positive (Figure 2A–H) and seven Gram-negative species (Figure 3A–G), among which two species, *Lachnospiraceae* bacterium 28–4 and *Firmicutes* bacterium ASF500, had not been separately cultivated *in vitro*. Most of the labeled Gram-positive bacteria divided in binary fission. *Lachnospiraceae* bacterium 28–4 (Figure 2A), *Clostridium clostridioforme* (Figure 2B) and an unclassified species of the *Fusicatenibacter* genus (Figure 2C), all of which belonged to the *Lachnospiraceae* family, divided by synthesis at the septum. As the most abundant species in this microbiota, *Lachnospiraceae* bacterium 28–4 was regarded as anti-inflammatory bacteria (Daniel et al., 2017). They were shown to increase the levels of polyamines, a group of compounds that were often associated with colon cancer and regulate inflammation by inhibiting butyrate production (Daniel et al., 2017). Using fluorescence microscopy, we found that this species had a slender spindle shape and elongated from many compact and discrete sections, presenting a unique two-color labeling feature. *C. clostridioforme* (Figure 2B), *Fusicatenibacter* (Figure 2C) and *Firmicutes* bacterium ASF500 (Figure 2D) presented dispersed growth during elongation and zonal synthesis for septation (Brown et al., 2011; Cava et al., 2013). *Clostridium difficile* (Figure 2E) and *Intestinimonas butyriciproducens* (Figure 2F) exhibited “medial” (also known as “pre-septal”) growth (Randich and Brun, 2015), where PGN was synthesized near the division plane before full assembly for septation. In *Eubacterium plexicaudatum* (Figure 2G) and *Acetivibrio ethanolgignens* (Figure 2H), FDAA-labeling signals indicated their dispersed mode of cell elongation (Cava et al., 2013). It is noteworthy that *A. ethanolgignens* (Figure 2H) had two highly overlapped FDAA-signals without an apparent septum, suggesting that they might have relatively stable cell wall synthesis rates when exposed to the two FDAA probes without going through any cell dividing.

Compared to Gram-positive bacteria, Gram-negative bacteria typically have a much thinner layer of PGN and exhibit relatively weak FDAA-labeling signals. *Bacteroides dorei* was labeled by FDAA in a special striped manner and divided in binary fission (Figure 3A). *Prevotella copri* was presented as short rods (∼1 μm in length) with a clearly growing septum in the midcell (Figure 3B). Several Gram-negative bacteria (Figures 3C–G) that taxonomically belonged to the *Clostridiales* order of the *Firmicutes* phylum showed comparable FDAA labeling intensities with Gram-positive bacteria. The thickness of the Gram-negative PGN in the trilaminar outer membrane was ∼8.5 nm (Cheng and Costerton, 1977), and Gram-positive’s PGN was ∼30–50 nm (Higgins and Shockman, 1970; McCready et al., 1976). *Butyrivibrio fibrisolvens*, which were normally stained as Gram-negative (Cheng and Costerton, 1977), presented a typical Gram-positive PGN labeling (Figure 3C). Their Gram-negative staining was probably because of the relatively thin cell walls (12∼18 nm), which could not retain the Gram-staining complex (Cheng and Costerton, 1977), but still thick enough to have a strong FDAA labeling. *Pseudoflavonifractor capillosus* (Figure 3D) was originally described as Gram-negative *Bacteroides capillosus* in the early twenties, and re-categorized as *Pseudoflavonifractor* that belonged to phylum *Firmicutes* in 2010, for its high genomic identity (>97%) with the *Flavonifractor* genus (Ricaboni et al., 2017). Belonging to the class *Clostridia*, which is usually identified as Gram-positive, *Hungatella* *Hathewayi* (Figure 3E) is, however, described as Gram-negative end-pointed bacilli (Steer et al., 2001). These three species (Figures 3C–E) elongated through dispersed lateral growth and divided with a prominent red-labeled septum in the midcell. Interestingly, some Gram-negative species belonging to the *Oscillibacter* genus (Figures 3F,G) of the phylum *Firmicutes* showed distinct labeling patterns with relatively strong intensities. *Oscillibacter ruminantium*, which could cause bacteremia (Sydenham et al., 2014), had asymmetric PGN synthesis (Figure 3F). *Oscillibacter valericigenes* (Broutin et al., 2020) exhibited a typical pre-septal elongation mode (Figure 3G).

### Snapshots of *Butyrivibrio fibrisolvens* Growing at Different Stages of a Cell Cycle.

Bacteria in various intestinal segments and niches were growing at distinct cellular stages during the STAMP labeling, and encountered the probes asynchronously. Therefore, it became possible to infer the growth stages of individual bacteria based on their two FDAA-labeling signals. Here, shown as an example, cells of *B. fibrisolvens* in different cell cycle stages were presented. As an important butyrate-producing rumen bacterium, *B. fibrisolvens* has been evaluated as a probiotic to prevent colorectal cancer (Ohkawara et al., 2005).

**FIGURE 4:**
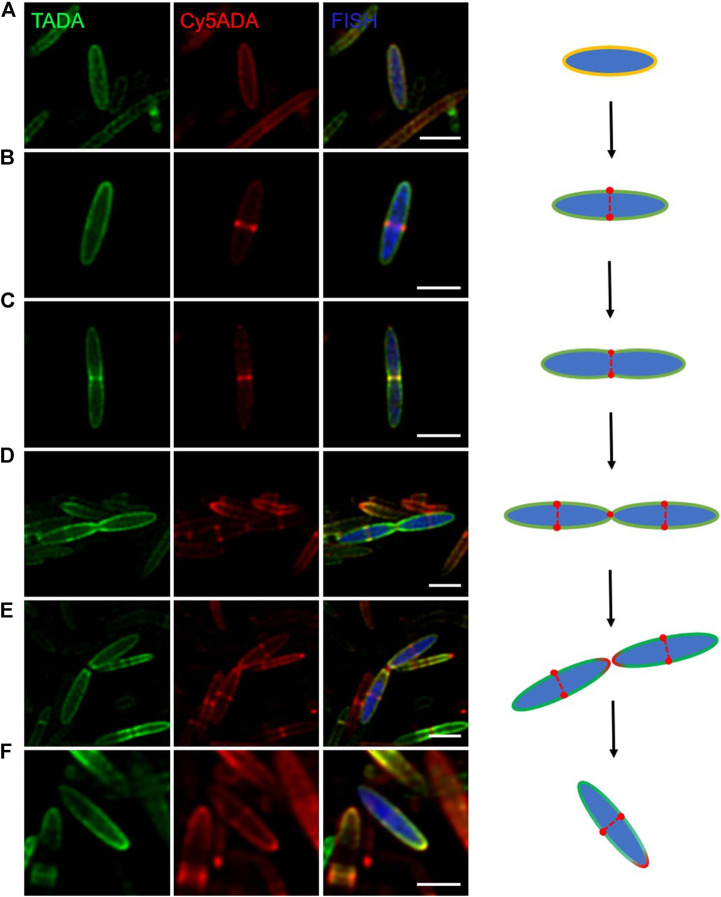
Confocal fluorescence imaging of *Butyrivibrio fibrisolvens* growing at different cell stages. Using FISH (blue) signals, cells of *B. fibrisolvens* sequentially labeled with TADA (green) and Cy5ADA (red) in the cecal microbiota were identified. **(A–F)** Confocal snapshots of six *B. fibrisolvens* cells at different growth stages were presented for a reconstructed cell cycle. Scale bars, 2 μm.

In the shown images of the two-color *B. fibrisolvens* (Figure 4), it can be observed that its life cycle involves primarily two classical growth modes: zonal cell wall synthesis and septum formation. Zonal cell wall synthesis means the incorporation of PGN along the sidewall, and septum formation denotes that the cytoskeletal division proteins direct the localization of PGN synthesis to generate nascent poles at the cell center (Brown et al., 2011; Randich and Brun, 2015). In a “first-generation” bacterium (∼3 µm in length), PGN was uniformly constructed along the lengthening cell (Figure 4A). When the cell elongated to ∼5 μm, an annular red-labeled incorporation zone appeared in the midcell, suggesting the beginning of septal formation (Figure 4B). With the continuous synthesis of PGN, *B. fibrisolvens* cell kept elongating until it reached twice the average length of a cell (Figures 4C,D). During division, the zonal growth site is modified to promote inwards growth of a septum and hydrolysis to form the new poles of the offspring cells (Figure 4E). Meanwhile, new growth regions were launched at the centers of the two daughter cells (Figures 4D,E). Then the daughter cells, which had only one red-labeled pole, continued to start the next cell cycle (Figure 4F). Through fine analysis of the STAMP labeling of individual cells, our method provides a unique angle to examine the basic microbial activities of specific bacterial species in the rat gut.

## Discussion

FDAAs have been a powerful and versatile tool for studying PGN synthesis (Hsu et al., 2017) owing to the efficient labeling of PGN in most bacterial species. Here, our STAMP + FISH strategy serves as an excellent platform to investigate microbial cytology in mammalian gut microbiotas, which could be impractical to approach by using other methods. Murine models have been continuously used in biomedical research for gut microbiota studies. Because of the compositions and diversities of bacterial species, researchers have been exploring the gut microbiotas of rat which show promise to become alternative to mouse microbiotas (Liou et al., 2013; Shao et al., 2017). Our labeling strategy offers new perspectives on bacterial activities that remain unknown, particularly those that have not been cultured *in vitro*. Furthermore, for some opportunistic pathogenic bacteria such as *C. difficile*, *I. butyriciproducens*, and *E. plexicaudatum*, their growing status and modes described here might provide insights for developing tailored therapeutic interventions. Of note, as an important opportunistic anaerobic pathogen, *C. difficile* is very rare in the gut of specific pathogen free mouse that we studied. Its relatively high abundance in the rat gut microbiota gives us an opportunity to study their growth and division in the mammalian gut, showcasing the importance of using STAMP to investigate gut bacteria in different hosts. The capability to differentiate bacteria of certain taxa growing at different stages, also offers an opportunity to better understand a complete cell cycle *in vivo*. Moreover, studies on gut microbiota of other mammalian hosts such as guinea pig (Hildebrand et al., 2012), pigs (Lamendella et al., 2011; Quan et al., 2020; Tang et al., 2020), dogs (Lin C. Y. et al., 2020), cats (Lyu et al., 2020), and primates such as chimpanzees (Moeller et al., 2016) and macaques (Manuzak et al., 2020), might also benefit by this integrative labeling techniques.

## Methods and Materials

### Reagents

The FDAA probes were synthesized by Chinese Peptide Company (Hangzhou, China). Stock solutions were prepared at concentrations of 10 mM in distilled H_2_O and stored at −20°C before use. FISH probes and paraformaldehyde fix solution were purchased from Sangon Biotech (Shanghai, China). Other chemicals, not mentioned above, were obtained from Sigma-Aldrich (St. Louis, MO, United states).

### Animals

Male Sprague-Dawley (SD) rats (weighing 220–250 g) were obtained from SLAC Laboratory Animal Corp (Shanghai, China). Rats were bred in the Department of Laboratory Animal Science of Fudan University under a 12 h day-night cycle at 25°C and a relative humidity of 50% for 7 days, receiving a standard chow diet and free access to clean water. All animal experiments were carried out in accordance with guidelines approved by the Department of Laboratory Animal Science and Use Committee of the Fudan University Institutes of Biomedical Sciences.

### Sequential Labeling of Rat Gut Microbiotas With Fluorescent D-amino Acid Probes

The SD rats were sequentially administered with two different FDAA probes (1 ml, 1 mM TADA or Cy5ADA in distilled H_2_O) through oral gavage with an interval of 3 h. Four hours after the second gavage, their cecal microbiotas were collected using a previously reported protocol (Lin L. et al., 2020). Briefly, rats were sacrificed after anesthetized excessively by intraperitoneal injections with 10% chloral hydrate, and the ceca were dissected separately and finely minced with a pair of 4.5-inch iris scissors in 6 ml of cold phosphate-buffered saline (PBS). The tissues and digesta were then filtered with a 40 μm cell strainer to remove most of the nonbacterial tissue debris. The filtrates were then centrifuged. The bacterial pellets (whitish-colored) were washed three times with 1.5 ml of PBS by centrifugation (15,000 g, 3 min) and then resuspended in PBS to reach an appropriate concentration for subsequent analyses by flow cytometry and confocal fluorescence microscopy.

### DNA Extraction and 16S rDNA Sequencing

DNA from the SD rat cecum’s bacterial samples was extracted using the Omega Bacterial DNA Kit (Omega Bio-Tek, Norcross, GA, United States) according to the manufacturer’s protocol. The V3–V4 hypervariable regions of the bacteria 16S rDNA were amplified by PCR and subsequently paired-end sequenced (2 × 300) on an Illumina MiSeq platform (Illumina, San Diego, United States) according to the standard protocols. The taxonomy of each 16S rRNA gene sequence was analyzed by RDP Classifier (http://rdp.cme.msu.edu/) against the SILVA (SSU123) 16S rDNA database with a confidence threshold of 80%.

### Metagenome Sequencing

Covaris M220 (Gene Company Limited, China) was used to construct the paired library with an average DNA fragment size of about 400 bp, and the paired library was sequenced on Illumina HiSeq4000 platform. BLASTP (Version 2.2.28+, http://blast.ncbi.nlm.nih.gov/Blast.cgi) was used for taxonomic annotations by aligning nonredundant gene catalogs against the integrated NR (non-redundant protein sequence) database with an e-value cutoff of 1 × 10^–5^.

### Fluorescence *in situ* Hybridization

Candidate FISH probes were designed using a k-mer–based algorithm similar to KASpOD (Parisot et al., 2012). The FDAA labeled microbiota was washed and resuspended in 50% paraformaldehyde-PBS (v/v) and incubated at room temperature for 1.5 h to fix the bacteria. After washed twice with PBS, an equal volume of EtOH was then added into the suspension and stored at −20°Cfor at least 48 h. The bacteria were spun down and resuspended in a hybridization buffer [0.9 M NaCl, 20 mM tris (pH, 7.5), 0.01% SDS, and formamide, if required]. FAM-labeled FISH probes were added to the sample with a final concentration of 5 ng/µl and incubated at 46°C for 4 h, using a ThermoMixer (Eppendorf, Hamburg, Germany). After hybridization, bacteria were washed two times (15 min) with washing buffer [0.9 M NaCl, 20 mM tris (pH 7.5), and 0.01% SDS], and then resuspended in Tris buffer [20 mM Tris and 25 mM NaCl (pH, 7.5)] before analysis with flow cytometry and fluorescence microscopy.

### Flow Cytometry

FDAA-labeled microbiota samples were analyzed using a CytoFLex flow cytometer (Beckman Coulter Life Sciences, Indianapolis, IN, United States). Data analyses were performed using FlowJo (V 10.0.8R1). The tagged microbiota was identified by flow cytometry of logFSC and logSSC, and then fluorescence gated. For each sample, 15,000 events were collected for analysis, with debris and dual particles excluded.

### Confocal Fluorescence Microscopy

The bacterial suspension was added to an agarose pad (1.5% in PBS, ∼1 mm thick), and the slides were covered with a glass cover slip. Confocal fluorescence imaging was performed using a TCS SP8 laser confocal microscope (Leica, Solms, Germany). Samples were excited for FAM (carboxyl fluorescein) at 488 nm, TAMRA at 555 nm, and Cy5 at 639 nm. The emission is detected by the corresponding emission filters. Image deconvolution was performed with Huygens Essential Deconvolution software (Scientific Volume Imaging, Hilversum, Netherlands), using a theoretical point expansion function.

## Data Availability

The 16S rDNA and shotgun sequencing data of the rat cecal microbiotas have been deposited in the Sequence Read Archive with BioSample accessions SAMN18356050 and SAMN14694443, respectively. Other relevant data related to this paper are available from the corresponding authors upon request.

## References

[B1] Boulangé C. L. Neves A. L. Chilloux J. Nicholson J. K. Dumas M.-E. (2016). Impact of the Gut Microbiota on Inflammation, Obesity, and Metabolic Disease. Genome Med. 8, 24. 10.1186/s13073-016-0303-2 27098727 PMC4839080

[B2] Broutin L. Deroche L. Michaud A. Le Moal G. Burucoa C. Gayet L.-E. (2020). First Description of Bacteremia Caused by *Oscillibacter Valericigenes* in a Patient Hospitalized for Leg Amputation. Anaerobe 64, 102244. 10.1016/j.anaerobe.2020.102244 32712374

[B3] Brown P. J. B. Kysela D. T. Brun Y. V. (2011). Polarity and the Diversity of Growth Mechanisms in Bacteria. Semin. Cel Dev. Biol. 22, 790–798. 10.1016/j.semcdb.2011.06.006 PMC3193591 21736947

[B4] Browne H. P. Forster S. C. Anonye B. O. Kumar N. Neville B. A. Stares M. D. (2016). Culturing of ʻunculturableʼ Human Microbiota Reveals Novel Taxa and Extensive Sporulation. Nature 533, 543–546. 10.1038/nature17645 27144353 PMC4890681

[B5] Cava F. Kuru E. Brun Y. V. de Pedro M. A. (2013). Modes of Cell Wall Growth Differentiation in Rod-Shaped Bacteria. Curr. Opin. Microbiol. 16, 731–737. 10.1016/j.mib.2013.09.004 24094807 PMC3931007

[B6] Cheng K. J. Costerton J. W. (1977). Ultrastructure of *Butyrivibrio Fibrisolvens*: a Gram-Positive Bacterium. J. Bacteriol. 129, 1506–1512. 10.1128/jb.129.3.1506-1512.1977 845122 PMC235129

[B7] Daniel S. G. Ball C. L. Besselsen D. G. Doetschman T. Hurwitz B. L. (2017). Functional Changes in the Gut Microbiome Contribute to Transforming Growth Factor β-Deficient Colon Cancer. mSystems 2, e00065-17. 10.1128/mSystems.00065-17 28951889 PMC5613170

[B8] Gill S. R. Pop M. Deboy R. T. Eckburg P. B. Turnbaugh P. J. Samuel B. S. (2006). Metagenomic Analysis of the Human Distal Gut Microbiome. Science 312, 1355–1359. 10.1126/science.1124234 16741115 PMC3027896

[B9] Higgins M. L. Shockman G. D. (1970). Model for Cell Wall Growth of *Streptococcus Faecalis* . J. Bacteriol. 101, 643–648. 10.1128/jb.101.2.643-648.1970 4984078 PMC284952

[B10] Hildebrand F. Ebersbach T. Nielsen H. Li X. Sonne S. Bertalan M. (2012). A Comparative Analysis of the Intestinal Metagenomes Present in guinea Pigs (*Cavia porcellus*) and Humans (*Homo sapiens*). BMC Genomics 13, 514. 10.1186/1471-2164-13-514 23020652 PMC3472315

[B11] Hsu Y.-P. Rittichier J. Kuru E. Yablonowski J. Pasciak E. Tekkam S. (2017). Full Color Palette of Fluorescentd-Amino Acids for In Situ Labeling of Bacterial Cell Walls. Chem. Sci. 8, 6313–6321. 10.1039/c7sc01800b 28989665 PMC5628581

[B12] Hudak J. E. Alvarez D. Skelly A. von Andrian U. H. Kasper D. L. (2017). Illuminating Vital Surface Molecules of Symbionts in Health and Disease. Nat. Microbiol. 2, 17099. 10.1038/nmicrobiol.2017.99 28650431 PMC5546223

[B13] Kong Y. He M. McAlister T. Seviour R. Forster R. (2010). Quantitative Fluorescence In Situ Hybridization of Microbial Communities in the Rumens of Cattle Fed Different Diets. Appl. Environ. Microbiol. 76, 6933–6938. 10.1128/aem.00217-10 20802069 PMC2953036

[B14] Kowluru R. A. (2020). Retinopathy in a Diet-Induced Type 2 Diabetic Rat Model and Role of Epigenetic Modifications. Diabetes 69, 689–698. 10.2337/db19-1009 31949005 PMC7085254

[B15] Kräker K. O’Driscoll J. M. Schütte T. Herse F. Patey O. Golic M. (2020). Statins Reverse Postpartum Cardiovascular Dysfunction in a Rat Model of Preeclampsia. Hypertension 75, 202–210. 10.1161/hypertensionaha.119.13219 31786987

[B16] Kuru E. Radkov A. Meng X. Egan A. Alvarez L. Dowson A. (2019). Mechanisms of Incorporation for D-Amino Acid Probes that Target Peptidoglycan Biosynthesis. ACS Chem. Biol. 14, 2745–2756. 10.1021/acschembio.9b00664 31743648 PMC6929685

[B17] Lamendella R. Santo Domingo J. W. Ghosh S. Martinson J. Oerther D. B. (2011). Comparative Fecal Metagenomics Unveils Unique Functional Capacity of the Swine Gut. BMC Microbiol. 11, 103. 10.1186/1471-2180-11-103 21575148 PMC3123192

[B18] Ley R. E. Hamady M. Lozupone C. Turnbaugh P. J. Ramey R. R. Bircher J. S. (2008). Evolution of Mammals and Their Gut Microbes. Science 320, 1647–1651. 10.1126/science.1155725 18497261 PMC2649005

[B19] Lin C.-Y. Cross T.-W. L. Doukhanine E. Swanson K. S. (2020). An Ambient Temperature Collection and Stabilization Strategy for Canine Microbiota Studies. Sci. Rep. 10, 13383. 10.1038/s41598-020-70232-6 32770113 PMC7414149

[B20] Lin L. Wu Q. Song J. Du Y. Gao J. Song Y. (2020). Revealing the In Vivo Growth and Division Patterns of Mouse Gut Bacteria. Sci. Adv. 6, eabb2531. 10.1126/sciadv.abb2531 32917613 PMC7473744

[B21] Liou A. P. Paziuk M. Luevano J.-M. Jr. Machineni S. Turnbaugh P. J. Kaplan L. M. (2013). Conserved Shifts in the Gut Microbiota Due to Gastric Bypass Reduce Host Weight and Adiposity. Sci. Transl. Med. 5, 178ra141. 10.1126/scitranslmed.3005687 PMC3652229 23536013

[B22] Lu K. H. Cao J. Oleson S. T. Powley T. L. Liu Z. (2017). Contrast-enhanced Magnetic Resonance Imaging of Gastric Emptying and Motility in Rats. IEEE Trans. Biomed. Eng. 64, 2546–2554. 10.1109/tbme.2017.2737559 28796602 PMC7439606

[B23] Lyu Y. Su C. Verbrugghe A. Van de Wiele T. Martos Martinez-Caja A. Hesta M. (2020). Past, Present, and Future of Gastrointestinal Microbiota Research in Cats. Front. Microbiol. 11, 1661. 10.3389/fmicb.2020.01661 32793152 PMC7393142

[B24] Manichanh C. Reeder J. Gibert P. Varela E. Llopis M. Antolin M. (2010). Reshaping the Gut Microbiome with Bacterial Transplantation and Antibiotic Intake. Genome Res. 20, 1411–1419. 10.1101/gr.107987.110 20736229 PMC2945190

[B25] Manuzak J. A. Zevin A. S. Cheu R. Richardson B. Modesitt J. Hensley-McBain T. (2020). Antibiotic-induced Microbiome Perturbations Are Associated with Significant Alterations to Colonic Mucosal Immunity in Rhesus Macaques. Mucosal. Immunol. 13, 471–480. 10.1038/s41385-019-0238-1 31797911 PMC7183431

[B26] Marchesi J. R. Adams D. H. Fava F. Hermes G. D. A. Hirschfield G. M. Hold G. (2016). The Gut Microbiota and Host Health: a New Clinical Frontier. Gut 65, 330–339. 10.1136/gutjnl-2015-309990 26338727 PMC4752653

[B27] McCready R. G. L. Costerton J. W. Laishley E. J. (1976). Morphological Modifications of Cells of *Clostridium Pasteurianum* Caused by Growth on Sulfite. Can. J. Microbiol. 22, 269–275. 10.1139/m76-036 1260530

[B28] Moeller A. H. Foerster S. Wilson M. L. Pusey A. E. Hahn B. H. Ochman H. (2016). Social Behavior Shapes the Chimpanzee Pan-Microbiome. Sci. Adv. 2, e1500997. 10.1126/sciadv.1500997 26824072 PMC4730854

[B29] Nicholson J. K. Holmes E. Kinross J. Burcelin R. Gibson G. Jia W. (2012). Host-gut Microbiota Metabolic Interactions. Science 336, 1262–1267. 10.1126/science.1223813 22674330

[B30] Ohkawara S. Furuya H. Nagashima K. Asanuma N. Hino T. (2005). Oral Administration of *Butyrivibrio Fibrisolvens*, a Butyrate-Producing Bacterium, Decreases the Formation of Aberrant Crypt Foci in the Colon and Rectum of Mice. J. Nutr. 135, 2878–2883. 10.1093/jn/135.12.2878 16317136

[B31] Parisot N. Denonfoux J. Dugat-Bony E. Peyret P. Peyretaillade E. (2012). KASpOD-A Web Service for Highly Specific and Explorative Oligonucleotide Design. Bioinformatics 28, 3161–3162. 10.1093/bioinformatics/bts597 23047560

[B32] Qin J. Li R. Li R. Raes J. Arumugam M. Burgdorf K. S. (2010). A Human Gut Microbial Gene Catalogue Established by Metagenomic Sequencing. Nature 464, 59–65. 10.1038/nature08821 20203603 PMC3779803

[B33] Quan J. Wu Z. Ye Y. Peng L. Wu J. Ruan D. (2020). Metagenomic Characterization of Intestinal Regions in Pigs with Contrasting Feed Efficiency. Front. Microbiol. 11, 32. 10.3389/fmicb.2020.00032 32038603 PMC6989599

[B34] Randich A. M. Brun Y. V. (2015). Molecular Mechanisms for the Evolution of Bacterial Morphologies and Growth Modes. Front. Microbiol. 6, 580. 10.3389/fmicb.2015.00580 26106381 PMC4460556

[B35] Ricaboni D. Mailhe M. Benezech A. Andrieu C. Fournier P.-E. Raoult D. (2017). ʻPseudoflavonifractor Phocaeensisʼ Gen. nov., Sp. nov., Isolated from Human Left Colon. New Microbes New Infect. 17, 15–17. 10.1016/j.nmni.2016.12.012 28275431 PMC5328723

[B36] Seluanov A. Gladyshev V. N. Vijg J. Gorbunova V. (2018). Mechanisms of Cancer Resistance in Long-Lived Mammals. Nat. Rev. Cancer 18, 433–441. 10.1038/s41568-018-0004-9 29622806 PMC6015544

[B37] Shao Y. Ding R. Xu B. Hua R. Shen Q. He K. (2017). Alterations of Gut Microbiota after Roux-En-Y Gastric Bypass and Sleeve Gastrectomy in Sprague-Dawley Rats. Obes. Surg. 27, 295–302. 10.1007/s11695-016-2297-7 27440168

[B38] Steer T. Collins M. D. Gibson G. R. Hippe H. Lawson P. A. (2001). *Clostridium Hathewayi* Sp. nov., from Human Faeces. Syst. Appl. Microbiol. 24, 353–357. 10.1078/0723-2020-00044 11822669

[B39] Sunde P. T. Olsen I. Göbel U. B. Theegarten D. Winter S. Debelian G. J. (2003). Fluorescence In Situ Hybridization (FISH) for Direct Visualization of Bacteria in Periapical Lesions of Asymptomatic Root-Filled Teeth. Microbiology (Reading) 149, 1095–1102. 10.1099/mic.0.26077-0 12724371

[B40] Sydenham T. V. Arpi M. Klein K. Justesen U. S. (2014). Four Cases of Bacteremia Caused by *Oscillibacter Ruminantium*, a Newly Described Species. J. Clin. Microbiol. 52, 1304–1307. 10.1128/jcm.03128-13 24501034 PMC3993470

[B41] Tang S. Xin Y. Ma Y. Xu X. Zhao S. Cao J. (2020). Screening of Microbes Associated with Swine Growth and Fat Deposition Traits across the Intestinal Tract. Front. Microbiol. 11, 586776. 10.3389/fmicb.2020.586776 33178171 PMC7596661

[B42] Tomas J. Langella P. Cherbuy C. (2012). The Intestinal Microbiota in the Rat Model: Major Breakthroughs from New Technologies. Anim. Health Res. Rev. 13, 54–63. 10.1017/s1466252312000072 22853927

[B43] Vuralli D. Wattiez A.-S. Russo A. F. Bolay H. (2019). Behavioral and Cognitive Animal Models in Headache Research. J. Headache. Pain 20, 11. 10.1186/s10194-019-0963-6 30704400 PMC6734244

[B44] Wos-Oxley M. L. Bleich A. Oxley A. P. A. Kahl S. Janus L. M. Smoczek A. (2012). Comparative Evaluation of Establishing a Human Gut Microbial Community within Rodent Models. Gut Microbes 3, 234–249. 10.4161/gmic.19934 22572831 PMC3427216

[B45] Zhang M. Liu Y. Zhao M. Tang W. Wang X. Dong Z. (2017). Depression and Anxiety Behaviour in a Rat Model of Chronic Migraine. J. Headache Pain 18, 27. 10.1186/s10194-017-0736-z 28224378 PMC5319946

[B46] Zhong J. Olsson L. M. Urbonaviciute V. Yang M. Bäckdahl L. Holmdahl R. (2018). Association of NOX2 Subunits Genetic Variants with Autoimmune Diseases. Free Radic. Biol. Med. 125, 72–80. 10.1016/j.freeradbiomed.2018.03.005 29526808

