# Supplementary material for: Visualizing the Growth and Division of Rat Gut Bacteria by D-Amino Acid-Based in vivo Labeling and FISH Staining
Source: Front Mol Biosci. 2021 May 28;8:681938. doi: 10.3389/fmolb.2021.681938 (PMC8193097; doi:10.3389/fmolb.2021.681938)
Supplement: Supplementary file 1 [file DataSheet2.PDF]

Supplementary Material

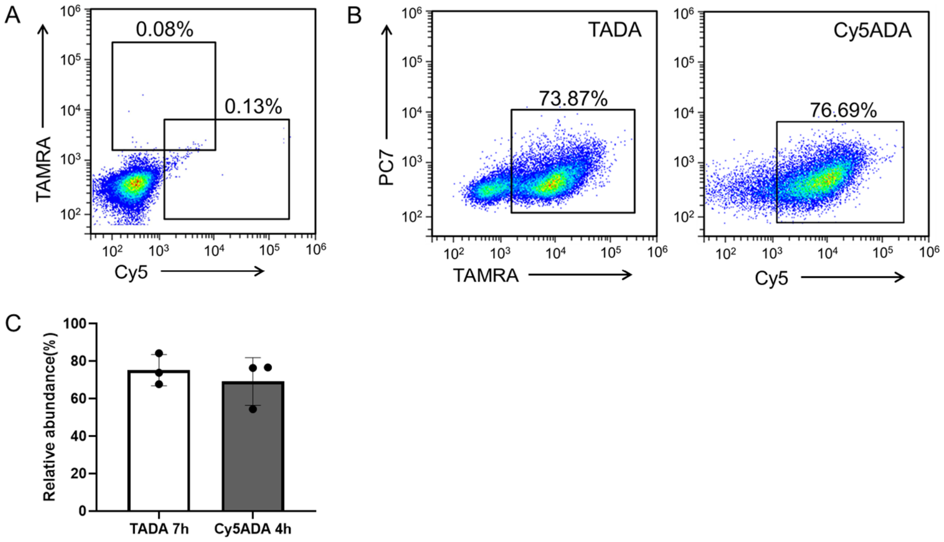

Fig. S1. Flow cytometry analysis of the STAMP-labeled rat gut microbiota. TADA and Cy5ADA were given to three rats by gavage sequentially at an interval of 3 h. Their cecal microbiotas were collected 4 h after the second gavage and analyzed by flow cytometry., Representative flow cytometry plots of the negative control (A) and STAMP-labeled (B) rat cecal microbiotas. (C)The labeling coverages of the two FDAA probes are comparable.

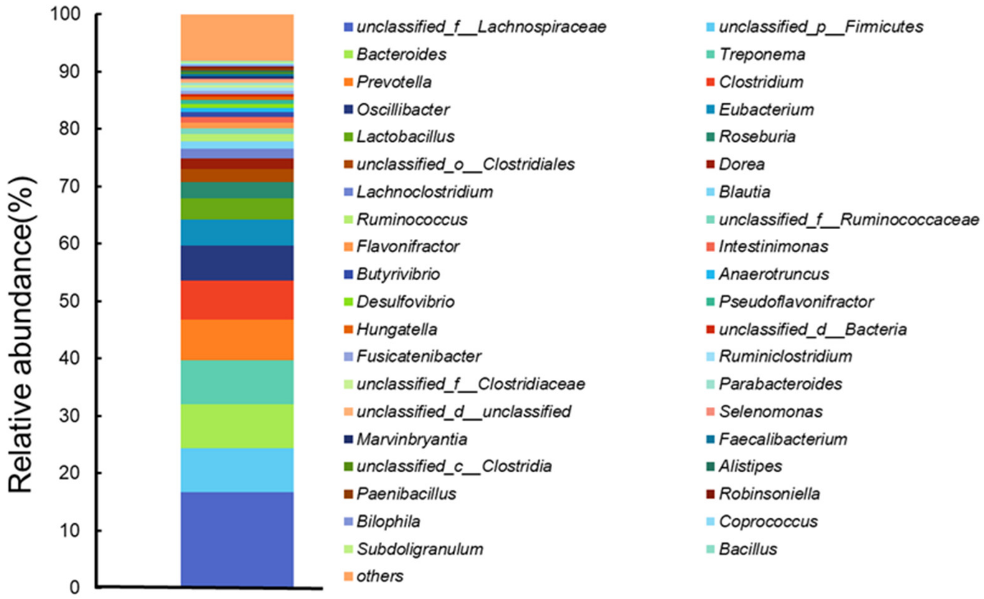

Fig. S2. Relative genus abundances of the cecal microbiota determined by 16s rDNA sequencing. Bacterial genera at levels of <0.2% are not displayed.

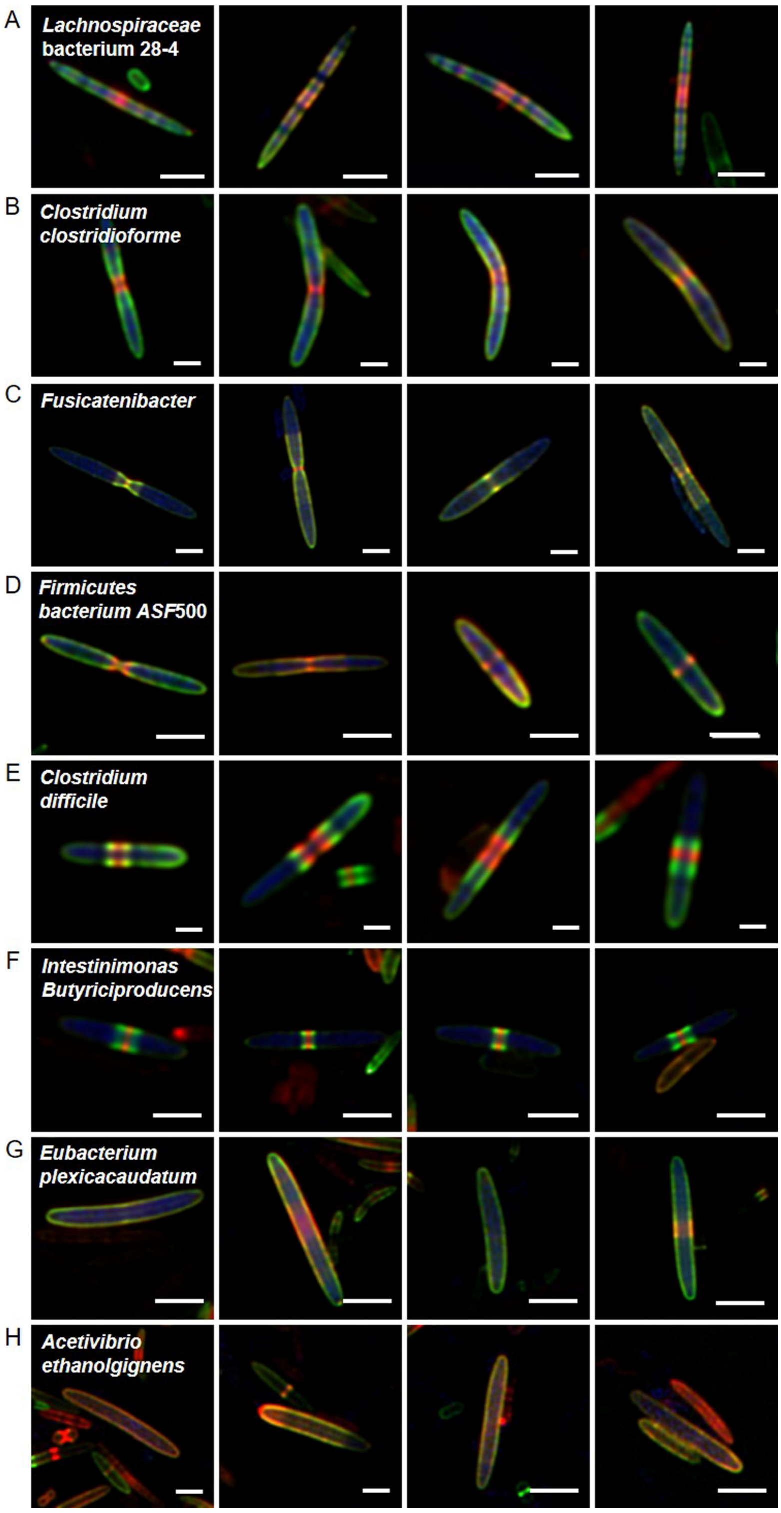
A
Lachnospiraceae bacterium 28-4
B
Clostridium clostridioforme
C
Fusicatenibacter
D
Firmicutes bacterium ASF500
E
Clostridium difficile
F
Intestinimonas Butyriciproducens
G
Eubacterium plexicacaudatum
H
Acetivibrio ethanolgignens

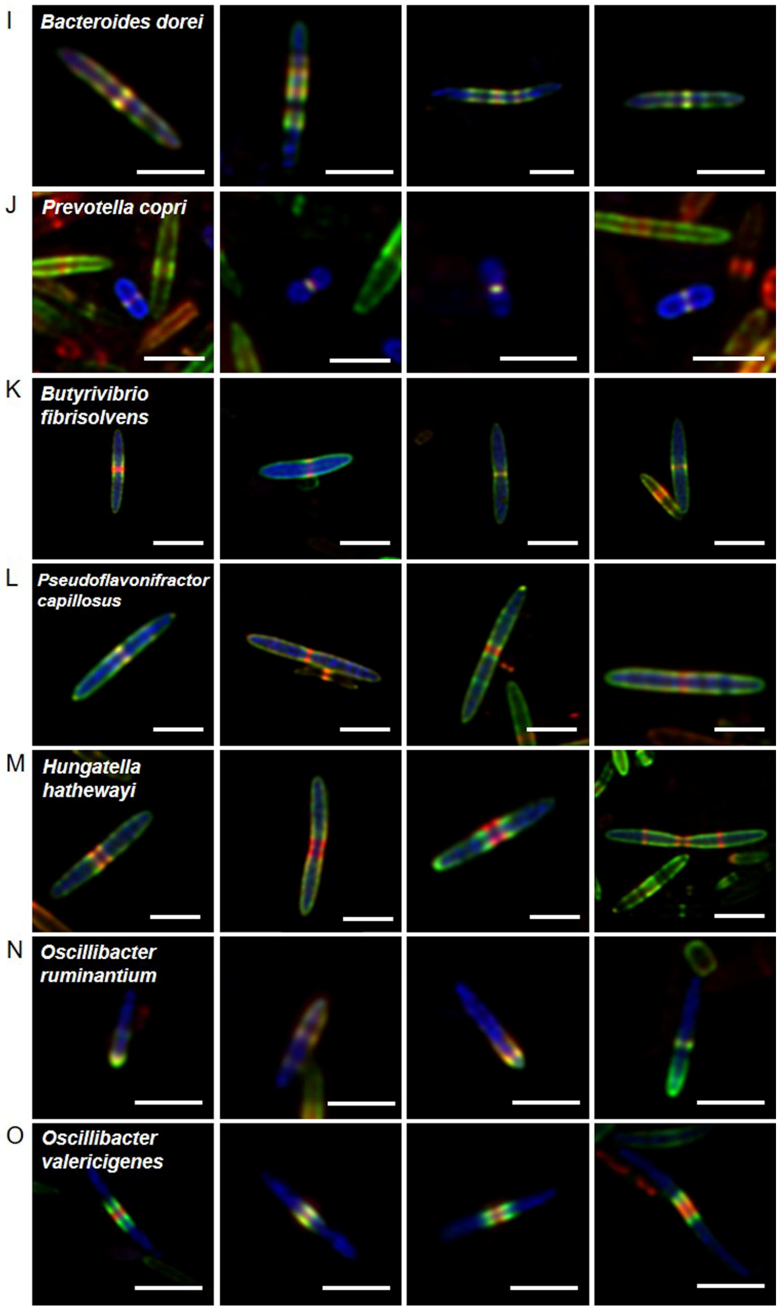

Fig. S3. Confocal fluorescence imaging of the STAMP-labeled bacteria showed consistent cell morphologies and FDAA-labeling patterns in each FISH-tagged species. The cecal microbiotas collected from rats received sequential labeling of TADA (green) and Cy5ADA (red) were stained by different FISH probes (blue) targeting corresponding species, and imaged by confocal fluorescence microscopy. Consistent labeling patterns and cell morphologies were observed in each species, including *Lachnospiraceae bacterium* 28-4 (A), *Clostridium clostridioforme* (B), *Fusicatenibacter* (C), *Firmicutes bacterium ASF*500 (D), *Clostridium difficile* (E), *Intestinimonas butyriciproducens* (F), *Eubacterium plexicacaudatum* (G), *Acetivibrio ethanolgignens* (H), *Bacteroides dorei* (I), *Prevotella copri* (J), *Butyrivibrio fibrisolvens* (K), *Pseudoflavonifractor capillosus* (L), *Hungatella hathewayi* (M), *Oscillibacter ruminantium* (N), and *Oscillibacter valericigenes* (O).

Table S2. The FISH probes used in this study.

Target Bacteria		Probe	Probe Sequence (5′–3′)	Hybridization temp (°C)	Formamide conc (%, v/v)	Source
Species	Family of Bacteria					
Lachnospiraceae bacterium 28-4	*Lachnospiraceae*	Lbc580	CGG CTG CCC CTC CCC TTC CGG GTT C	46	20	This study
Clostridium clostridioforme	*Lachnospiraceae*	Ccl633	AGG GCT TTG CCC CCC AAC ACC TAG C	46	35	This study
Butyrivibrio fibrisolvens	*Lachnospiraceae*	Bfi628	ATG GCA CCC AAC ACC TAG	46	20	(1)
Fusicatenibacter	*Lachnospiraceae*	Fus300	AGT AGC CGG CGT GAG AGG GCG AC	50	20	This study
Firmicutes bacterium ASF500	*Clostridiaceae*	Fba995	CGG CGC TTC CGA AGA AGA AAG TGT A	46	20	This study
Hungatella hathewayi	*Clostridiaceae*	Chath1100	CTC CCC AGA GTG CCC GAC TCT ACT C	46	20	This study
Intestinimonas butyriciproducens	*Intestinimonas*	Ibu1240	GGG ACA GCT TTT AGG GAT TTG CT	46	20	This study
Eubacterium plexicaudatum	*Eubacterium*	Epl765	ACG CCA TTT CTG GGG TTT GCT CCA C	46	20	This study
Acetivibrio ethanolgignens	*Ruminococcaceae*	Aet87	AGT CAA ATA AAA CTT CTC TTC CT	50	30	This study
Clostridium difficile	*Peptostreptococcaceae*	Cdi929	GAT GTC ATT GGG ATG TCA AGC TT	46	20	This study
Pseudoflavonifractor capillosus	*Pseudoflavonifractor*	Pca806	CTG CGG CAA CGG AGG GGG TCA GT	46	30	This study
Prevotella copri	*Prevotellaceae*	Pco1436	GTT TCG CCC TAG GCC GCT CCT TAC G	46	20	This study
Bacteroides dorei	*Bacteroidaceae*	Bdo162	TCA TGA TGC CAT CCT GGA TTA ATC T	46	30	This study
Oscillibacter ruminantium	*Oscillospiraceae*	Oru5	TCA AGG GTG TTC CGT TCG ACT GCA T	46	20	This study
Oscillibacter valericigenes	*Oscillospiraceae*	Ova193	TCA AGC GAT GTC GCC CAA ATG TAT C	46	35	This study

Table S3. FISH probes failed to be confirmed of their specificities.

Target Bacteria		Probe	Probe Sequence (5′–3′)	Hybridization temp (°C)	Formamide conc (%, v/v)	Source
Species	Family of Bacteria					
Turicibacter sanguinis	*Erysipelotrichaceae*	Tsa1429	ATC ATC TAC CCC ACC TTA GGC AGG CT CCG	46	20	This study
Fusicatenibacter saccharivorans	*Lachnospiraceae*	Fsa965	TTA CGG GCC GGT CAT CGG GA	46	30	This study
Tyzzerella nexilis	*Lachnospiraceae*	Tne977	CCC ATT ACT GGA CCG GTC ACC GG	46	20	This study
Flavonifractor plautii	*Clostridiales*	Fcl583	AAT GCA GGC TGG AGG TTG AGC CCC CAG T	46	20	This study
Bacteroides sartorii	*Bacteroidaceae*	Bsa131	GAG AAA ATG CTG GCC CGT GAT GCC A	46	20	This study
Oscillibacter sp. ER4	*Oscillospiraceae*	OER1201	TTG GGG GTT TGC TCT GCC TCG CGG C	46	20	This study
Bacteroides pyogenes	*Bacteroidaceae*	Bpy54	TCA AAT ATT GGC AAG CCA ATA TTC A	46	20	This study
Prevotella brevis	*Prevotellaceae*	Ppy65	CTT CAA AAG CAA GCT TTC GAA ATG A	46	20	This study
Lactobacillus sp. ASF360	*Lactobacillaceae*	LAS1352	CTC CTT CCA CAT AAG TGG TTA GGC C	46	20	This study
Lactobacillus johnsonii	*Lactobacillaceae*	Ljo446	ACC TCT ATC TTT CTT CAC TAC CAA C	46	20	This study

1. Kong Y, He M, McAlister T, Seviour R, Forster R. Quantitative fluorescence in situ hybridization of microbial communities in the rumens of cattle fed different diets. Appl. Environ. Microbiol. 2010, 76: 6933.
